# An Exploratory Study of Heat Shock Protein Changes in Women with Unexplained Infertility

**DOI:** 10.3390/ijms27114817

**Published:** 2026-05-27

**Authors:** Zainab Alhalwachi, Thozhukat Sathyapalan, Alexandra E. Butler, Stephen L. Atkin

**Affiliations:** 1 School of Medicine, Royal College of Surgeons in Ireland, Medical University of Bahrain, Busaiteen 15503, Bahrain; zalhalwachi@rcsi.com (Z.A.); satkin@rcsi.com (S.L.A.); 2 Hull York Medical School, University of Hull, Hull HU6 7RX, UK; thozhukat.sathyapalan@hyms.ac.uk

**Keywords:** HSP70, HSC70, STIP1, unexplained infertility, in vitro fertilization

## Abstract

Unexplained infertility affects up to 30% of couples and has been associated with heat shock proteins (HSP) and endometrial stress. HSPs and their co-chaperones are part of a complex network of proteins responsible for maintaining protein homeostasis and cell survival. This exploratory hypothesis-generating study investigated the possible relationship between HSPs and unexplained infertility. Twenty-five women were recruited from an IVF clinic. Eleven were confirmed for unexplained infertility (UI), while fourteen were age- and body mass index (BMI)-matched couples with confirmed male factor infertility (MFI), acting as controls. Blood samples were obtained at day 21 of the luteal phase, and plasma measurement of 19 HSPs and co-chaperones undertaken using the slow off-rate modified aptamer (SomaScan) platform. Welch’s *t*-test and a permutation test were used to compare group means, and Pearson’s correlations to examine relationships with HSPs. Of the 19 proteins measured, plasma HSP70 was decreased (permutation *p* = 0.002) in cases with unexplained infertility, while HSC70 and STIP1 were increased (permutation *p* = 0.017 and *p* = 0.001, respectively) when compared to MFI control. HSP70 was negatively correlated to both HSC70 and STIP 1 in UI (r = −0.77, permutation *p* = 0.017; −0.80, permutation *p* = 0.003, respectively), but not in MFI, whilst HSC70 and STIP1 were positively correlated in both UI and MFI (r = 0.93, permutation *p* = 0.001; r = 0.65, permutation *p* = 0.035, respectively). The HSP70-HSC70-STIP1 axis showed HSC70-STIP1 coupling with an inverse relationship with inducible HSP70, findings that may suggest dysregulation of constitutive and stress-inducible chaperone systems in UI.

## 1. Introduction

Infertility is defined as failing to conceive within 12 consecutive months of unprotected sexual activity [1] and affects about 17.5% of adults globally [2]. Causes of infertility can be due to male or female factors or a combination of both. Female factors of infertility include physical issues such as tubal occlusion, endometriosis and endometrial polyps/fibroids [3]. Ovulatory dysfunction results in infertility through disorders such as polycystic ovarian syndrome (PCOS) and obesity [3]. Male infertility stems from conditions such as varicocele, low sperm count, and poor sperm quality (motility, morphology, and DNA integrity). These are influenced by hormonal and genetic factors, as well as modifiable lifestyle behaviors—obesity disrupts endocrine balance and raises scrotal temperature, smoking induces oxidative DNA damage, and alcohol suppresses testosterone production [4].

Infertility has been linked to poor oocyte quality [5], luteinized unruptured follicle syndrome [6], sperm function deficits [7], defects in endometrial receptivity [8] and endometriosis [9], among several other factors including lifestyle [10,11]. The diagnosis of UI can only be made as a diagnosis of exclusion when all known causes of infertility are eliminated [12].

Heat shock protein 70 (HSP70) has been shown to be increased in infertility, with the suggestion that the heat shock response (HSR) may be involved in unexplained infertility [13]. The HSR is a cellular protective pathway activated in response to stressors such as extreme heat, oxidative stress, inflammation, or hypoxia [14]. HSR is mediated by heat shock proteins (HSPs) and their co-chaperones, which maintain proteostasis through regulation of protein folding stabilization, trafficking, and degradation [15]. Under proteotoxic stress, heat shock factor 1 (HSF1) dissociates from inhibitory chaperone complexes, trimerizes, translocates to the nucleus, and binds heat shock elements (HSEs) in the promoters of HSP genes to drive transcription of cytoprotective chaperones such as HSP70 and HSP90 [16]. HSPs are increasingly recognized as important regulators of endometrial function and embryo–maternal interactions, with dysregulation of HSPs being linked to implantation failure and infertility [17,18,19]. In addition, HSPs have been linked to male infertility (HSP27, 60, 70, 90) PCOS (HSP70, 90) and spontaneous abortions (HSP70, HSC70) [20,21,22,23,24]. The HSPs and their co-chaperones reflect a complex interactive network; therefore, this exploratory hypothesis-generating study aimed to determine a wide spectrum of these proteins and their differential expression in women with UI compared to control women with male factor infertility (MFI).

## 2. Results

### 2.1. Patient Characteristics

Results from all metabolic and hormonal analyses and IVF treatment can be found in Table 1. There were no differences found between the MFI and UI groups for any of the metabolites measured. Similarly, no differences were seen between MFI and UI for any of the hormonal parameters measured. Results from the in vitro fertilization (IVF) parameters showed no significant differences between the number of positive pregnancy tests, eggs retrieved or embryos created.

### 2.2. Heat Shock Proteins and Co-Chaperones Measurements

Of the 19 proteins investigated, 7 were major chaperone HSPs: heat shock protein 27 (HSP27), heat shock protein 40 (HSP40), heat shock protein 60 (HSP60), heat shock protein 70 (HSP70), heat shock cognate protein 70 (HSC70), heat shock protein 90 alpha/beta (HSP90a/b), and heat shock protein 90 beta (HSP90b). Another 5 proteins were co-chaperone proteins: cell division cycle 37 (CDC37), peptidyl-prolyl cis-trans isomerase D (PPID), stress-induced phosphoprotein 1 (STIP1), carboxyl terminus of Hsp70-interacting protein (CHIP), and clusterin. The remaining 7 proteins play various roles in the cellular stress response networks. Toll-like receptor 4 (TLR4) and toll-like receptor 4 in complex with myeloid differentiation factor-2 (TLR4:MD-2) are immune receptor components. MAP kinase-activated protein kinase 2 (MAPK2) and MAP kinase-activated protein kinase 5 (MAPK5) are signaling kinases. Ubiquitin-conjugating enzyme E2 G2 (UB2G2) is a ubiquitin-conjugating enzyme. Endothelial monocyte-activating polypeptide 2 (EMAP-2) is a pro-inflammatory cytokine. Calcineurin is a phosphatase involved in cellular signaling. All results are shown in Table 2.

**Figure 1:**
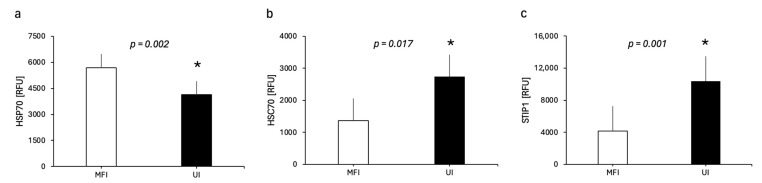
Expression levels of heat shock protein: (**a**) HSP70 was significantly decreased (*p* = 0.003) in UI patients, (**b**) HSC70 was significantly increased (*p* = 0.017) in UI compared to MFI, (**c**) co-chaperone protein STIP1 was significantly increased (*p* = 0.019) in UI. Protein levels are reported as relative fluorescence units (RFU). * Permutation test *p* < 0.05.

The mean expression level of plasma HSP70 was significantly decreased in the UI group compared to the MFI control group with a large effect size as measured by Welch’s *t*-test (*p* = 0.003, Cohen’s d = −1.36) and a permutation test (*p* = 0.002). There was also a significant increase in the mean expression level of plasma STIP1 in UI with a large effect size as measured by Welch’s *t*-test (*p* = 0.019, Cohen’s d = 1.02) and the permutation test (*p* = 0.001) (Figure 1). Additionally, HSC70 was significantly increased in UI as measured by the permutation test (*p* = 0.017) but was not significant when measured by Welch’s *t*-test (*p* = 0.131). Nine proteins showed medium effects (|d| 0.5–0.8; HSP90A/B, HSP90b, CDC37, PPID, UB2G2, MAPK2, MAPK5, TLR4, TLR4:MD-2 but these did not reach statistical significance (sensitivity analysis using the Grubbs test confirmed that the results for HSP70, HSC70 and STIP1 were outlier-independent (Supplementary Table S1)). There were no significant differences amongst other heat shock proteins or co-chaperone proteins.

### 2.3. Correlation Analysis

A correlation analysis was carried out for significant proteins HSP70, HSC70 and STIP1 with vitamin D3, AMH, G3D3, fertilization rate, top quality embryo (proportion), FSH and LH. There were no significant correlations.

As seen in Table 3 when compared to MFI control, HSP70 was negatively correlated with both HSC70 and STIP 1 in UI (r = −0.77, permutation *p* = 0.017; r = −0.80, permutation *p* = 0.003, respectively), but not in MFI, whilst HSC70 and STIP1 were positively correlated in both UI and MFI (r = 0.93, permutation *p* = 0.001; r = 0.65, permutation *p* = 0.012, respectively). These findings are shown as a scatter plot in Supplementary Figure S1.

**Figure 2:**
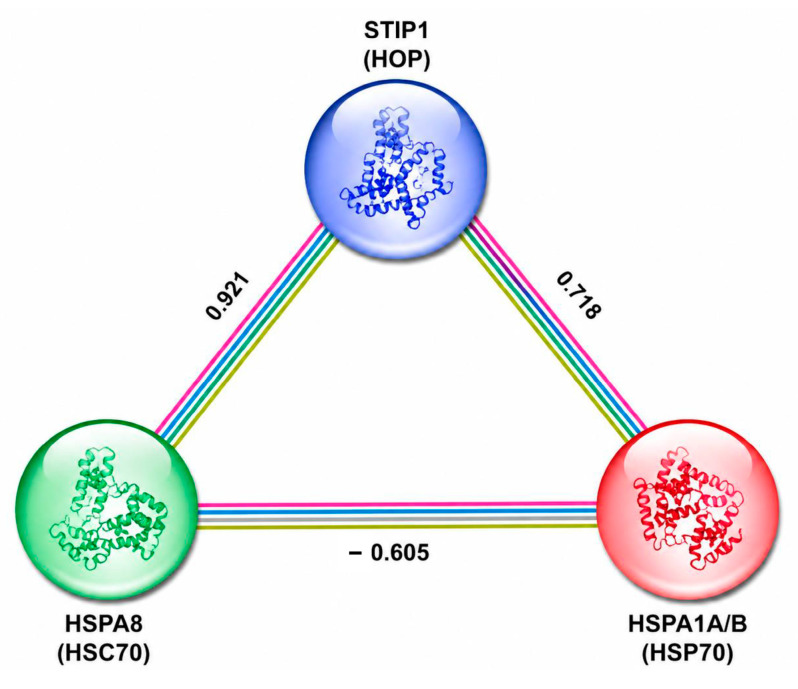
STRING-style network illustrating pairwise correlations between HSP70 (HSPA1A/B), HSC70 (HSPA8), and STIP1 (HOP) across the study cohort. Nodes represent proteins, and edges denote Pearson correlation coefficients derived from quantitative proteomic data. Edge labels indicate correlation strength (r values), with positive associations reflecting coordinated expression and negative associations indicating inverse regulation.

String analysis for HSP70 (HSPA1A/B), HSC70 (HSPA8), and STIP1 (HOP) is shown in Figure 2.

## 3. Discussion

This study provides a quantitative proteomic comparison of heat shock protein (HSP) and co-chaperone expression profiles between patients with UI and MFI. The circulating plasma heat shock and other related proteins may be composite results reflecting systemic protein activity as well as proteins derived from tissues, including the endometrium. Therefore, the results here may represent a combination of systemic and localized tissue-derived responses. We identified two proteins with significant, outlier-independent differential expression: HSP70 (HSPA family; elevated in MFI) and STIP1 (elevated in UI). These findings survived a sensitivity analysis using the Grubbs test. Several further proteins in the HSP90 co-chaperone network demonstrated consistent directional trends warranting investigation in adequately powered studies.

HSP70 expression was significantly increased in the MFI relative to UI group. HSP70 plays a critical role in multiple cellular functions including reproduction, where it is expressed in reproductive tissues such as the endometrium and uterus [25]. Inflammation has been linked to elevated HSP70 expression [26]. In this study, there was no significant increase in CRP in the UI or MFI group, suggesting that there was not a systemic overt inflammatory response, though CRP alone cannot exclude local, low-grade, reproductive-tract, or tissue-specific inflammatory processes. The reduced plasma HSP70 levels observed in the UI may reflect altered systemic stress-response activity associated with oxidative stress in reproductive complications, which can damage or change the protein’s conformation and make it ineffective [27,28,29], although the present study did not assess protein functionality or tissue-specific effects. Additionally, circulating HSP70 levels may be associated with broader stress-related reproductive processes [30,31], including apoptotic activity that was not evaluated in the present study. Elevated total HSP70 signals in MFI plasma hypothetically may reflect chaperone upregulation secondary to proteotoxic stress [32]. Our finding of higher HSP70 in the MFI group at the whole-sample proteomic level is consistent with this model, though it is important to note that the current dataset does not discriminate between individual HSPA isoforms, and the relative contributions of HSPA1A, HSPA1B and HSPA2 cannot be resolved from the protein identifiers used. Future studies employing isoform-specific mass spectrometry or targeted immunoassay will be required to determine whether the elevated signal reflects predominantly HSPA2 or the inducible HSPA1A/B species. While our study shows dysregulation of circulating levels of HSP70, interpretations regarding tissue-specific reproductive mechanisms should be considered exploratory with further investigations required to assess their validity.

A statistically significant and biologically compelling finding was the markedly higher STIP1 expression in the UI group. STIP1(HOP) serves as a critical scaffold that physically transfers client proteins from HSP70 to HSP90, coordinating the sequential chaperoning of client proteins including steroid hormone receptors [33]. Overexpression of STIP1 is associated with poor blastocyst development in human IVF trials and has been implicated in infertility [34]. HSP90 is critical for the correct folding and stabilization of the progesterone receptor (PR) and estrogen receptor-α (ERα) [35]; impairment of the HSP70–STIP1–HSP90 relay results in receptor ubiquitination and degradation, compromising endometrial differentiation during the implantation window [36,37]. Upregulation of STIP1 may therefore represent a compensatory response to increased load on the HSP90 system, potentially arising from oxidative or proteotoxic stress in the endometrium of UI patients.

UBE2G2 showed a medium negative effect (d = −0.72) with nominally elevated expression in MFI that approached but did not reach uncorrected significance (Welch’s *p*  =  0.069). UBE2G2 is the principal E2 enzyme partnering with the CHIP/STUB1 E3 ubiquitin ligase complex to target HSP70- and HSP90-bound misfolded client proteins for proteasomal degradation [38]. A larger study is needed to investigate this further.

HSC70 (HSPA8) displayed a divergence between parametric and non-parametric test results. Although Welch’s *t*-test yielded *p* = 0.131, the permutation test returned *p* = 0.017; the divergence can be explained by extreme variance heterogeneity in the UI group (SD = 3295 vs. MFI SD = 206), driven largely by an outlier sample. The outlier inflated both the UI mean and its variance, paradoxically worsening the Welch statistic while not affecting the permutation test, which operates on rank order. After outlier exclusion in the sensitivity analysis, UI variance reduced substantially (SD = 899) and the effect size strengthened (d = +0.69 from +0.63), lending credence to a genuine difference. HSC70 is constitutively expressed and participates in the endoplasmic reticulum-associated degradation (ERAD) pathway [39]; its elevation in UI may hypothetically reflect increased ER stress in endometrial tissue, consistent with published transcriptomic data showing upregulation of the unfolded protein response in endometrium from patients with recurrent pregnancy loss [40].

The STRING-based network analysis provided complementary, systems-level validation of the correlation findings, showing that the HSP70–HSC70–STIP1 axis forms a highly interconnected module rather than a set of isolated associations. The high-confidence interaction between HSC70 and STIP1, supported by both experimental and curated database evidence in STRING, aligns with the very strong positive correlation observed in our dataset (r = 0.921), thus reinforcing the biological validity of this axis. Importantly, the network topology suggests that STIP1 functions as a critical integrative node between these proteins.

Several HSP90 co-chaperones and client-network proteins showed medium effect sizes in the UI direction (HSP90AB1 d = +0.62; CDC37 d = +0.56; MAPKAPK2 d = +0.58; MAPKAPK5 d = +0.55; PPID d =  +0.53) that did not individually reach statistical significance, likely reflecting insufficient statistical power at the current sample size. Power calculations indicate that detection of medium effects (d ≈ 0.5–0.6) at α = 0.05 and 1 − β = 0.80 requires approximately 50–60 participants per group under Welch’s framework. These proteins form a functionally coherent cluster: CDC37 is the dedicated co-chaperone for HSP90-mediated kinase maturation [41], while MAPKAPK2 and MAPKAPK5 are among its principal client kinases, involved in the p38 MAPK stress-response cascade [42]. Potentially the elevated HSP90 network activity in UI is consistent with the hypothesis that heightened proteostatic demand, arising from immune dysregulation, oxidative stress, or subtle endometrial pathology, may drive compensatory upregulation of the HSP90 chaperoning system.

The TLR4 complex (TLR4 and its co-receptor MD-2/LY96) showed medium-sized effects in opposing directions that were stable across outlier exclusion; however, there was no differences between MFI and UI, which may be a reflection of sample size and should be addressed in a further larger study. TLR4 itself trended higher in MFI (d = −0.53; MFI  >  UI), while TLR4/LY96 co-receptor expression favored MFI expression (d = −0.56). TLR4 is a pattern-recognition receptor activated by HSP60 and HSP70 acting as damage-associated molecular patterns (DAMPs), constituting a key mechanism by which extracellular HSPs trigger innate immune responses at the endometrial surface [43]. Studies have reported elevated TLR4 expression in the endometrium of women with miscarriages [44]. Whilst these findings were not significant here, they warrant repeating in a larger cohort.

A strength of the study was that all samples were taken at the same time in the menstrual cycle and the state-of-the-art proteomic analysis. However, the study has several limitations. First, the sample sizes (UI *n*  =  11, MFI *n*  =  14 following outlier identification) are modest, resulting in limited statistical power to detect medium effect sizes after multiple comparison correction. The BH-FDR procedure, while appropriate for controlling the false discovery rate across 19 proteins, was conservative under these sample constraints: even HSP70 and STIP1, with the smallest uncorrected *p*-values, did not survive FDR correction (q  =  0.068 and 0.40 respectively). Second, the proteomic platform used provides composite protein-level intensities and cannot resolve between closely related isoforms, as discussed for the HSPA family, and measurement of protein levels from plasma may not represent tissue levels. Third, potential confounding factors, including BMI, metabolic status and low-grade inflammation, may influence chaperone expression and were not fully adjusted in all analyses due to the small sample size. Fourth, this cross-sectional study cannot establish causality; whether altered HSP expression is a cause or consequence of the infertility phenotype remains to be determined through longitudinal and interventional designs. Although the women in the MFI group underwent clinical evaluation to exclude female-factor infertility, subclinical endometrial or ovarian pathology cannot be fully excluded. All subjects were Caucasian and therefore the study needs to be repeated with an ethnically diverse population to determine the generalizability of the study. Diet may affect HSPs, and dietary counseling is routinely given to all patients with IVF, but formal dietary assessment was not conducted in this study.

## 4. Materials and Methods

### 4.1. Study Design and Participants

In this cross-sectional case-control study, all 25 Caucasian participants as a group were recruited sequentially from an in vitro fertilization (IVF) unit in Hull, UK, over a 4-month period. Due to the exploratory nature of the study, no power calculation was carried out and the study population of 25 was determined based on participant availability and the inclusion/exclusion criteria applied. All participants were women of reproductive age (20–45 years) with a body mass index (BMI) of <30 kg/m^2^ and were undergoing IVF treatment. The study population were women diagnosed with UI, in accord with recognized guidelines [12] that included diagnostic laparoscopy. The control population were healthy women with MFI [12], where the men were diagnosed as infertile [45,46]. Patients were excluded from the study if they had a record of smoking, alcohol consumption or were taking medications, whether prescription or over-the-counter therapies, within the last six months. Patients were also excluded if they had documented immunological or inflammatory disease, acute or chronic infection, hepatic insufficiency or renal insufficiency. Ethical approval was granted by the Yorkshire and the Humber NRES Ethics Committee, UK (approval number 02/03/043) [47].

### 4.2. Sample Collection and IVF Treatment

Fasting blood samples were collected at day 21 of the menstrual cycle for all participants, at which time mock embryo transfer was undertaken before the start of the IVF treatment; ultrasound confirmed the presence of a corpus luteum at that visit. The samples were centrifuged at 3500× *g* for 15 min at 4 °C before storing the plasma at −80 °C until further analysis. All participants underwent IVF treatment during their next menstrual cycle as previously described [48]. The IVF protocol has been described previously [48] and embryo transfer was carried out on day 3 or preferably day 5 (blastocyst stage) to maximize implantation potential. Embryo quality was assessed at both cleavage and blastocyst stages using standardized morphological grading criteria [49].

### 4.3. Metabolic Measures

Total cholesterol, triglyceride, high-density lipoprotein cholesterol (HDL-c), C-reactive protein (CRP) and fasting blood glucose (FBG) were measured using a Synchron LX20 analyzer (Beckman Coulter, High Wycombe, UK). White blood cell (WBC) count was measured using a Beckman Coulter counter (Beckman Coulter). Low-density lipoprotein cholesterol (LDL-c) was calculated using the Friedewald equation [50]. Insulin concentrations were analyzed using a competitive chemiluminescent immunoassay (Immulite 2000, Euro/DPC, Llanberis, UK), and insulin resistance was assessed by the homeostatic model assessment of insulin resistance (HOMA-IR), calculated as (insulin × glucose)/22.5 [51]. Glycated hemoglobin (HbA1c) was detected using ion-exchange chromatography.

### 4.4. Reproductive Hormone Quantification

To determine anti-Müllerian hormone (AMH) levels, an immunoenzymatic assay (Beckman Coulter) was used. Circulating androgens were quantified by liquid chromatography–tandem mass spectrometry (LC/MS/MS; Acquity UPLC-Quattro Premier XE-MS, Waters, Manchester, UK). Sex hormone-binding globulin (SHBG) was assessed using an immunometric fluorescence assay (Immulite 2000 analyzer; upper limit 2.0 nmol/L). The free androgen index (FAI) was calculated according to the formula (testosterone/SHBG) × 100. Thyroid hormone levels, thyroid stimulating hormone (TSH), free T3, and free thyroxine (Free-T4) were determined using an immunoassay on the Abbott Architect i4000 platform (Abbott Diagnostics, Maidenhead, UK).

### 4.5. Heat Shock Proteins and Co-Chaperone Expression

Heat shock proteins were quantified using the slow off-rate modified aptamer (SomaScan) platform, version 3.1 (SomaLogic, Boulder, CO, USA), as previously described in detail [52,53]. Nineteen heat shock proteins were investigated in this study: heat shock protein 90 alpha/beta (HSP 90a/b), heat shock protein 90 beta (HSP90b), heat shock protein 70 (HSP70), heat shock protein family A member 8/heat shock cognate protein 70 (HSPA8/HSC70), heat shock protein 27 (HSP27/HSPB1), heat shock protein 60 (HSP60), cell division cycle 37 (CDC37), heat shock protein 40 (HSP40), peptidyl-prolyl cis-trans isomerase D (PPID), stress-induced phosphoprotein 1 (STIP1), carboxyl terminus of Hsp70-interacting protein (CHIP), ubiquitin-conjugating enzyme E2 G2 (UB2G2), endothelial monocyte-activating polypeptide 2 (EMAP-2), clusterin, MAP kinase-activated protein kinase 2 (MAPK2), MAP kinase-activated protein kinase 5 (MAPK5), calcineurin, toll-like receptor 4 (TLR4), toll-like receptor 4 in complex with myeloid differentiation factor-2 (TLR4:MD-2).

STRING 12.0 (Search Tool for the Retrieval of Interacting Genes, https://string-db.org, accessed 18 April 2026) was used to visualize the known and predicted protein–protein interactions (PPIN) for proteins that were dysregulated.

While the SomaScan platform is a state-of-the-art proteomic analysis, it is unable to differentiate between HSP isoforms.

### 4.6. Statistical Analysis

No formal power calculation was performed due to the exploratory nature of this pilot study; however, *n* = 25 was chosen to estimate effect sizes for future studies. Continuous data is presented as mean ± standard deviation (SD). Welch’s *t*-test was used to compare the mean differences between test groups, and *p* < 0.05 was considered statistically significant. Due to the small sample size, a permutation test was used with 10,000 permutations and *p* < 0.05 was considered significant. Effect sizes (Cohen’s d) were calculated using both pooled and unequal-variance standardization, yielding consistent interpretations. FDR correction was applied across all 19 Welch *p*-values for the primary group comparison. To examine the relationship between heat shock proteins and co-chaperones and metabolic and hormonal measurements, pairwise correlations between HSP70 (HSPA1A/B), HSC70 (HSPA8), and STIP1 (HSP70/HSP90-organising protein (HOP)) were calculated using two-tailed Pearson correlation coefficients (r) derived from log-transformed protein expression values. Statistical significance was assessed using corresponding *p* values, with adjustment for multiple comparisons performed using the Benjamini–Hochberg false discovery rate (FDR) method. Sensitivity analysis was undertaken using the Grubbs test. Correlation networks were visualized as a STRING-style node–edge diagram, with edge weights proportional to correlation strength and annotated with r values. All statistical analyses and graphs were carried out using GraphPad Prism v.9.5.1 (San Diego, CA, USA).

## 5. Conclusions

In conclusion, this exploratory pilot study identifies differential plasma expression and altered correlation structure of the HSP70–HSC70–STIP1 axis in women with unexplained infertility compared with women undergoing IVF for male factor infertility. Although findings did not remain significant after correction across the full protein panel and require validation, the large effect size for HSP70 and STIP1, together with altered chaperone-network coupling, supports further investigation of proteostasis and stress-adaptation pathways in unexplained infertility.

## Figures and Tables

**Table 1:** Demographic, biochemical, hormonal, and IVF parameter data compared between male factor infertility (MFI) and unexplained infertility (UI).

	MFI (*n* = 14) Mean (SD)	UI (*n* = 11) Mean (SD)	*p* Value
Age (years)	32.6 (4.0)	33.8 (5.3)	0.51
BMI (kg/m^2^)	25.7 (2.6)	25.3 (4.9)	0.84
HOMA-IR	1.5 (0.7)	1.9 (1.4)	0.45
Cholesterol (mmol/L)	4.8 (0.8)	4.6 (0.7)	0.44
Triglycerides (mmol/L)	0.9 (0.5)	1.0 (0.4)	0.67
HDL-c (mmol/L)	1.6 (0.4)	1.6 (0.2)	0.97
LDL-c (mmol/L)	2.8 (0.7)	2.7 (0.6)	0.24
CRP (mg/L)	1.9 (1.3)	2.4 (2.0)	0.51
WBC 10^9^/L	5.9 (1.7)	7.2 (2.2)	0.12
AMH (ng/mL)	22.4 (15.3)	24.5 (12.5)	0.72
FAI	1.4 (0.7)	0.8 (0.9)	0.11
TSH (mU/L)	2.3 (1.2)	2.0 (0.8)	0.53
Free-T3 (pmol/L)	4.7 (0.7)	4.8 (0.6)	0.66
Free-T4 (pmol/L)	11.2 (1.6)	11.4 (0.9)	0.66
Positive pregnancy test	0.3 (0.5)	0.3 (0.5)	0.95
Number of eggs retrieved	9.0 (7.5)	8.4 (3.2)	0.78
Number of embryos created	3.7 (3.0)	5.2 (2.4)	0.18
G3D3	3.4 (2.2)	2.7 (2.6)	0.49
Fertility rate	0.6 (0.2)	0.6 (0.4)	0.89
Top quality embryo (proportion)	0.3 (0.2)	0.4 (0.4)	0.36

BMI, body mass index; HOMA-IR, Homeostatic Model Assessment of Insulin Resistance; HDL-c, High-Density Lipoprotein Cholesterol; LDL-c, Low-Density Lipoprotein Cholesterol; CRP, C-Reactive Protein; WBC, white blood cell count; AMH, Anti-Müllerian Hormone; FAI, Free Androgen Index; TSH, thyroid-stimulating hormone; Free-T3, Free-triiodothyronine; Free-T4, Free-Thyroxine; and G3D3, Top-Quality Day 3 Embryos (Grade 3, Day 3).

**Table 2:** Heat shock protein levels reported as relative fluorescence units (RFUs) in male factor infertility (MFI) and unexplained infertility (UI).

	MFI (*n* = 14) Mean (SD)	UI (*n* = 11) Mean (SD)	*p* Value (*t*-Test)	*p* Value (perm.)	Cohen’s d	95% CI	
						Lower Limit	Upper Limit
HSP27	2533 (2284)	2098 (1068)	0.566	0.611	−0.23	−1.03	0.56
HSP40	382 (103)	509 (457)	0.323	0.409	0.41	−0.39	1.2
HSP60	3181 (2609)	3378 (3950)	0.882	0.860	0.06	−0.73	0.85
HSP70	5686 (1050)	4126 (1260)	0.003 *	0.002 *	−1.36	−2.24	−0.49
HSC70	1365 (206)	2738 (3296)	0.131	0.017 *	0.63	−0.18	1.44
HSP90a/b	3232 (855)	8469 (15876)	0.228	0.064	0.50	−0.3	1.3
HSP90b	23,173 (6381)	38,318 (36,498)	0.139	0.056	0.62	−0.19	1.43
CDC37	332 (113)	413 (175)	0.178	0.190	0.57	−0.24	1.36
PPID	1507 (887)	2904 (3831)	0.198	0.152	0.53	−0.27	1.34
STIP1	4150 (1402)	10,380 (9145)	0.019 *	0.001 *	1.02	0.18	1.86
CHIP	1250 (1595)	969 (295)	0.572	0.960	−0.23	−1.02	0.56
UB2G2	4758 (1741)	3750 (792)	0.089	0.087	−0.72	−1.53	0.1
EMAP-2	3068 (706)	2856 (716)	0.466	0.481	−0.30	−1.09	0.5
Clusterin	830 (77)	886 (253)	0.441	0.576	0.32	−0.48	1.11
MAPK2	1821 (833)	2757 (2242)	0.161	0.163	0.58	−0.22	1.39
MAPK5	500 (179)	610 (224)	0.186	0.179	0.55	−0.26	1.35
Calcineurin	594 (209)	1459 (2887)	0.273	0.234	0.45	−0.35	1.25
TLR4	377 (327)	243 (70)	0.198	0.213	−0.54	−1.34	0.27
TLR4:MD-2	3939 (850)	3449 (895)	0.175	0.173	−0.56	−1.37	0.24

HSP 90a/b, heat shock protein 90-alpha/beta; HSP90b, heat shock protein 90-beta; HSP70, heat shock protein 70; HSC70, heat shock cognate protein 70; HSP27, heat shock protein 27; HSP60, heat shock protein 60; CDC37, cell division cycle 37; HSP40, heat shock protein 40; PPID, peptidyl-prolyl cis-trans isomerase D; STIP1, stress-induced phosphoprotein 1; CHIP, carboxyl terminus of Hsp70-interacting protein; UB2G2, ubiquitin-conjugating enzyme E2 G2; EMAP-2, endothelial monocyte-activating polypeptide 2; MAPK2, MAP kinase-activated protein kinase 2; MAPK5, MAP kinase-activated protein kinase 5; perm, permutation test; *t*-test, Welch’s *t*-test; TLR4, toll-like receptor 4; TLR4:MD-2, toll-like receptor 4 in complex with myeloid differentiation factor-2. * *p* < 0.05.

**Table 3:** Pearson correlation coefficients among heat shock protein 70 (HSP70), heat shock cognate protein 70 (HSC70), and stress-induced phosphoprotein 1 (STIP1) in patients with unexplained infertility (UI) and male factor infertility (MFI).

Group	Pair	Pearson r	Pearson *p*	Permutation *p*	FDR q (Pearson)	FDR q (Permutation)
UI	HSP70 vs. HSC70	−0.77	0.005	0.017	0.005	0.017
UI	HSP70 vs. STIP1	−0.80	0.002	0.003	0.004	0.006
UI	HSC70 vs. STIP1	0.93	0.00003	0.001	0.00009	0.003
MFI	HSP70 vs. HSC70	0.20	0.489	0.50	0.489	0.503
MFI	HSP70 vs. STIP1	−0.38	0.170	0.175	0.256	0.262
MFI	HSC70 vs. STIP1	0.65	0.011	0.012	0.035	0.037

## Data Availability

The original contributions presented in this study are included in the article/Supplementary Materials. Further inquiries can be directed to the corresponding author.

## Supplementary Materials

The following supporting information can be downloaded at: https://www.mdpi.com/article/10.3390/ijms27114817/s1.

## References

[1] Nugent C.N., Chandra A. (2024). Infertility and Impaired Fecundity in Women and Men in the United States, 2015–2019.

[2] Wei Y., Lin Z., Huang Q., Wu H., Wang R., Wang J. (2025). Burden of female infertility in 204 countries and territories, 1990-2021: Results from the Global Burden of Disease Study 2021. J. Psychosom. Obstet. Gynaecol..

[3] Carson S.A., Kallen A.N. (2021). Diagnosis and management of infertility: A review. JAMA.

[4] Eisenberg M.L., Esteves S.C., Lamb D.J., Hotaling J.M., Giwercman A., Hwang K., Cheng Y.S. (2023). Male infertility. Nat. Rev. Dis. Primers.

[5] Homer H.A. (2020). The Role of Oocyte Quality in Explaining “Unexplained” Infertility. Semin. Reprod. Med..

[6] Qublan H., Amarin Z., Nawasreh M., Diab F., Malkawi S., Al-Ahmad N., Balawneh M. (2006). Luteinized unruptured follicle syndrome: Incidence and recurrence rate in infertile women with unexplained infertility undergoing intrauterine insemination. Hum. Reprod..

[7] Schattman G.L., Esteves S.C., Agarwal A. (2015). Unexplained infertility. Female Reproductive Dysfunction.

[8] Santamaria X., Simón C. (2021). Endometrial factor in unexplained infertility and recurrent implantation failure. Seminars in Reproductive Medicine.

[9] Van Gestel H., Bafort C., Meuleman C., Tomassetti C., Vanhie A. (2024). The prevalence of endometriosis in unexplained infertility: A systematic review. Reprod. Biomed. Online.

[10] Biggs S.N., Kennedy J., Lewis S.L., Hearps S., O’Bryan M.K., McLachlan R., von Saldern S., Chambers G., Halliday J. (2023). Lifestyle and environmental risk factors for unexplained male infertility: Study protocol for Australian Male Infertility Exposure (AMIE), a case–control study. Reprod. Health.

[11] Anderson K., Nisenblat V., Norman R. (2010). Lifestyle factors in people seeking infertility treatment–a review. Aust. N. Z. J. Obstet. Gynaecol..

[12] Romualdi D., Ata B., Bhattacharya S., Bosch E., Costello M., Gersak K., Homburg R., Mincheva M., Norman R., Guideline Group on Unexplained Infertility (2023). Evidence-based guideline: Unexplained infertility. Hum. Reprod..

[13] Nip M.M., Miller D., Taylor P.V., Gannon M.J., Hancock K.W. (1994). Expression of heat shock protein 70 kDa in human endometrium of normal and infertile women. Hum. Reprod..

[14] Sisti G., Kanninen T.T., Ramer I., Witkin S.S. (2015). Interaction between the inducible 70-kDa heat shock protein and autophagy: Effects on fertility and pregnancy. Cell Stress Chaperones.

[15] Singh M.K., Shin Y., Ju S., Han S., Choe W., Yoon K.-S., Kim S.S., Kang I. (2024). Heat shock response and heat shock proteins: Current understanding and future opportunities in human diseases. Int. J. Mol. Sci..

[16] Barna J., Csermely P., Vellai T. (2018). Roles of heat shock factor 1 beyond the heat shock response. Cell. Mol. Life Sci..

[17] Brosens J.J., Salker M.S., Teklenburg G., Nautiyal J., Salter S., Lucas E.S., Steel J.H., Christian M., Chan Y.-W., Boomsma C.M. (2014). Uterine selection of human embryos at implantation. Sci. Rep..

[18] Berestoviy V., Mahmood A., Venckivska I., Ginzburg V., Sokol I., Berestoviy O., Govsieiev D. (2021). The overview and role of heat shock proteins (HSP) especially HSP 60 and 70 in reproduction and other pathologies (a literature review). Meдичні Пepcпeктиви.

[19] Swelum A.A.-A., Hashem N.M., Abo-Ahmed A.I., Abd El-Hack M.E., Abdo M. (2020). The role of heat shock proteins in reproductive functions. Heat Shock Proteins in Inflammatory Diseases.

[20] Purandhar K., Jena P.K., Prajapati B., Rajput P., Seshadri S. (2014). Understanding the role of heat shock protein isoforms in male fertility, aging and apoptosis. World J. Men’s Health.

[21] Feng H.L., Sandlow J.I., Sparks A.E. (2001). Decreased expression of the heat shock protein hsp70-2 is associated with the pathogenesis of male infertility. Fertil. Steril..

[22] Niinuma S.A., Lubbad L., Lubbad W., Moin A.S.M., Butler A.E. (2023). The role of heat shock proteins in the pathogenesis of polycystic ovarian syndrome: A review of the literature. Int. J. Mol. Sci..

[23] Wu G., Hu X., Ding J., Yang J. (2019). Abnormal expression of HSP70 may contribute to PCOS pathology. J. Ovarian Res..

[24] Gulic T., Laskarin G., Dominovic M., Glavan Gacanin L., Babarovic E., Haller H., Rukavina D. (2016). Potential role of heat-shock protein 70 and interleukin-15 in the pathogenesis of threatened spontaneous abortions. Am. J. Reprod. Immunol..

[25] Jee B., Dhar R., Singh S., Karmakar S. (2021). Heat Shock Proteins and Their Role in Pregnancy: Redefining the Function of “Old Rum in a New Bottle”. Front. Cell Dev. Biol..

[26] Molvarec A., Rigó J., Lázár L., Balogh K., Makó V., Cervenak L., Mézes M., Prohászka Z. (2009). Increased serum heat-shock protein 70 levels reflect systemic inflammation, oxidative stress and hepatocellular injury in preeclampsia. Cell Stress Chaperones.

[27] Grunwald M.S., Pires A.S., Zanotto-Filho A., Gasparotto J., Gelain D.P., Demartini D.R., Schöler C.M., de Bittencourt P.I.H., Moreira J.C.F. (2014). The oxidation of HSP70 is associated with functional impairment and lack of stimulatory capacity. Cell Stress Chaperones.

[28] Agarwal A., Aponte-Mellado A., Premkumar B.J., Shaman A., Gupta S. (2012). The effects of oxidative stress on female reproduction: A review. Reprod. Biol. Endocrinol..

[29] Polak G., Rola R., Gogacz M., Kozioł-Montewka M., Kotarski J. (1999). Malonyldialdehyde and total antioxidant status in the peritoneal fluid of infertile women. Ginekol. Pol..

[30] Ravagnan L., Gurbuxani S., Susin S.A., Maisse C., Daugas E., Zamzami N., Mak T., Jäättelä M., Penninger J.M., Garrido C. (2001). Heat-shock protein 70 antagonizes apoptosis-inducing factor. Nat. Cell Biol..

[31] Jurisicova A. (1998). Programmed Cell Death During Mammalian Preimplantation Embryo Development, Genetic Regulation and Developmental Consequences. Ph.D. Thesis.

[32] Deka K., Saha S. (2018). Regulation of mammalian HSP70 expression and stress response. Regulation of Heat Shock Protein Responses.

[33] Bhattacharya K., Weidenauer L., Luengo T.M., Pieters E.C., Echeverría P.C., Bernasconi L., Wider D., Sadian Y., Koopman M.B., Villemin M. (2020). The Hsp70-Hsp90 co-chaperone Hop/Stip1 shifts the proteostatic balance from folding towards degradation. Nat. Commun..

[34] McReynolds S., Dzieciatkowska M., Stevens J., Hansen K.C., Schoolcraft W.B., Katz-Jaffe M.G. (2014). Toward the identification of a subset of unexplained infertility: A sperm proteomic approach. Fertil. Steril..

[35] Dhamad A.E., Zhou Z., Zhou J., Du Y. (2016). Systematic Proteomic Identification of the Heat Shock Proteins (Hsp) that Interact with Estrogen Receptor Alpha (ERα) and Biochemical Characterization of the ERα-Hsp70 Interaction. PLoS ONE.

[36] Smith D.F., Toft D.O. (2008). Minireview: The intersection of steroid receptors with molecular chaperones: Observations and questions. Mol. Endocrinol..

[37] Tranguch S., Cheung-Flynn J., Daikoku T., Prapapanich V., Cox M.B., Xie H., Wang H., Das S.K., Smith D.F., Dey S.K. (2005). Cochaperone immunophilin FKBP52 is critical to uterine receptivity for embryo implantation. Proc. Natl. Acad. Sci. USA.

[38] Smith C.E., Tsai Y.C., Liang Y.H., Khago D., Mariano J., Li J., Tarasov S.G., Gergel E., Tsai B., Villaneuva M. (2021). A structurally conserved site in AUP1 binds the E2 enzyme UBE2G2 and is essential for ER-associated degradation. PLoS Biol..

[39] Liu T., Daniels C.K., Cao S. (2012). Comprehensive review on the HSC70 functions, interactions with related molecules and involvement in clinical diseases and therapeutic potential. Pharmacol. Ther..

[40] Tang T., Fu J., Zhang C., Wang X., Cao H., Chen L. (2024). Exploring the role of endoplasmic reticulum stress in recurrent spontaneous abortion: Identification of diagnostic biomarkers and immune cell interactions. Heliyon.

[41] Oberoi J., Guiu X.A., Outwin E.A., Schellenberger P., Roumeliotis T.I., Choudhary J.S., Pearl L.H. (2022). HSP90-CDC37-PP5 forms a structural platform for kinase dephosphorylation. Nat. Commun..

[42] Moens U., Kostenko S., Sveinbjørnsson B. (2013). The Role of Mitogen-Activated Protein Kinase-Activated Protein Kinases (MAPKAPKs) in Inflammation. Genes.

[43] Vabulas R.M., Ahmad-Nejad P., Ghose S., Kirschning C.J., Issels R.D., Wagner H. (2002). HSP70 as endogenous stimulus of the Toll/interleukin-1 receptor signal pathway. J. Biol. Chem..

[44] Kolben T.M., Rogatsch E., Hester A., Kuhn C., Schmoeckel E., Czogalla B., Mahner S., Jeschke U., Kolben T. (2019). Involvement of ILR4α and TLR4 in miscarriages. J. Reprod. Immunol..

[45] Schlegel P.N., Sigman M., Collura B., De Jonge C.J., Eisenberg M.L., Lamb D.J., Mulhall J.P., Niederberger C., Sandlow J.I., Sokol R.Z. (2021). Diagnosis and treatment of infertility in men: AUA/ASRM guideline part I. Fertil. Steril..

[46] Schlegel P.N., Sigman M., Collura B., De Jonge C.J., Eisenberg M.L., Lamb D.J., Mulhall J.P., Niederberger C., Sandlow J.I., Sokol R.Z. (2021). Diagnosis and treatment of infertility in men: AUA/ASRM guideline part II. Fertil. Steril..

[47] Malik H., Zamouri S., Akkawi S., Mehra S., Mouaki R., Sathyapalan T., Nandakumar M., Butler A.E., Atkin S.L. (2025). Endothelial Protein Changes Indicative of Endometriosis in Unexplained Infertility, an Exploratory Study. Int. J. Mol. Sci..

[48] Brennan E., Radhi M.K., Husain Z.A., Sathyapalan T., Moin A.S.M., Butler A.E., Atkin S.L. (2025). Differential Expression of Complement Pathway Components in Unexplained Infertility Versus Male Factor Infertility: Insights from an Exploratory Pilot Study. Int. J. Mol. Sci..

[49] Medicine A.S.i.R. (2012). The Alpha consensus meeting on cryopreservation key performance indicators and benchmarks: Proceedings of an expert meeting. Reprod. Biomed. Online.

[50] Friedewald W.T., Levy R.I., Fredrickson D.S. (1972). Estimation of the concentration of low-density lipoprotein cholesterol in plasma, without use of the preparative ultracentrifuge. Clin. Chem..

[51] Matthews D.R., Hosker J.P., Rudenski A.S., Naylor B., Treacher D.F., Turner R. (1985). Homeostasis model assessment: Insulin resistance and β-cell function from fasting plasma glucose and insulin concentrations in man. Diabetologia.

[52] Gold L., Ayers D., Bertino J., Bock C., Bock A., Brody E., Carter J., Cunningham V., Dalby A., Eaton B. (2010). Aptamer-based multiplexed proteomic technology for biomarker discovery. PLoS ONE.

[53] Suhre K., Arnold M., Bhagwat A.M., Cotton R.J., Engelke R., Raffler J., Sarwath H., Thareja G., Wahl A., DeLisle R.K. (2017). Connecting genetic risk to disease end points through the human blood plasma proteome. Nat. Commun..

