# Supplementary material for: An Exploratory Study of Heat Shock Protein Changes in Women with Unexplained Infertility
Source: Int J Mol Sci. 2026 May 27;27(11):4817. doi: 10.3390/ijms27114817 (PMC13256705; doi:10.3390/ijms27114817)
Supplement: Supplementary file 1 [file ijms-27-04817-s001.zip › ijms-4321290-supplementary.pdf]

Supplementary table S1

Grubbs test for sensitivity

	Sensitivity analysis (UI n = 10, outlier excluded)						Comparison	
**Protein**	**UI mean ± SD**	**t statistic**	**p (Welch)**	**p (BH-FDR)**	**p (perm)**	**Cohen's d (excl.)**	**Δ Cohen's d**	**Δ |%|**
**HSP70**	4,258.3 ± 1,105.2	-3.198	**0.0041**	0.0684	**0.0038**	**-1.295**	+0.067	4.9%
**STIP1**	8,056.1 ± 3,862.4	2.407	**0.0440**	0.4036	**0.0036**	**+1.113**	+0.095	9.3%
UBE2G2	3,762.4 ± 812.1	-1.875	0.0726	0.4036	0.1057	-0.704	+0.011	1.5%
HSC70	1,974.3 ± 899.1	1.420	0.1849	0.4036	**0.0498**	+0.693	+0.063	10.0%
HSP90AB1	33,104.2 ± 22,640.1	1.241	0.2416	0.4036	0.2083	+0.549	-0.068	11.0%
MAPKAPK2	2,284.1 ± 1,108.3	0.821	0.4310	0.4036	0.4148	+0.355	-0.228	39.1%
TLR4 LY96	3,512.8 ± 921.3	-0.988	0.3365	0.4036	0.3409	-0.395	+0.169	30.0%
CDC37	388.4 ± 139.2	0.845	0.4062	0.4036	0.4780	+0.319	-0.241	43.0%
MAPKAPK5	598.1 ± 212.4	1.200	0.2439	0.4036	0.2150	+0.525	-0.024	4.4%
TLR4	246.9 ± 71.2	-1.322	0.1833	0.4036	0.2792	-0.493	+0.041	7.7%
PPID	2,301.3 ± 1,614.8	0.723	0.4759	0.4036	0.4707	+0.302	-0.232	43.4%
HSP90AA1	6,821.4 ± 9,204.2	1.008	0.3307	0.4036	0.2971	+0.445	-0.054	10.8%
PPP3CA/R1	538.1 ± 196.2	-0.040	0.9684	0.9684	0.9642	-0.017	**-0.470**	**103.8%**
DNAJB1	376.5 ± 107.6	-0.053	0.9574	0.9574	0.9522	-0.024	**-0.431**	**105.9%**
CLU	818.2 ± 71.4	-0.573	0.6483	0.6483	0.6387	**-0.197**	**-0.513**	**162.3%**
AIMP1	2,894.1 ± 698.3	-0.126	0.9017	0.9017	0.9113	-0.047	+0.251	**84.2%**
HSPB1	2,086.4 ± 982.1	-1.011	0.3322	0.3322	0.4461	-0.361	-0.127	54.3%
STUB1	972.4 ± 302.1	-0.625	0.5413	0.5413	0.9569	-0.220	+0.011	4.8%
HSPD1	2,714.3 ± 1,188.4	-1.313	0.1993	0.1993	0.2964	**-0.477**	**-0.538**	**882.0%**

Supplementary Figure S1

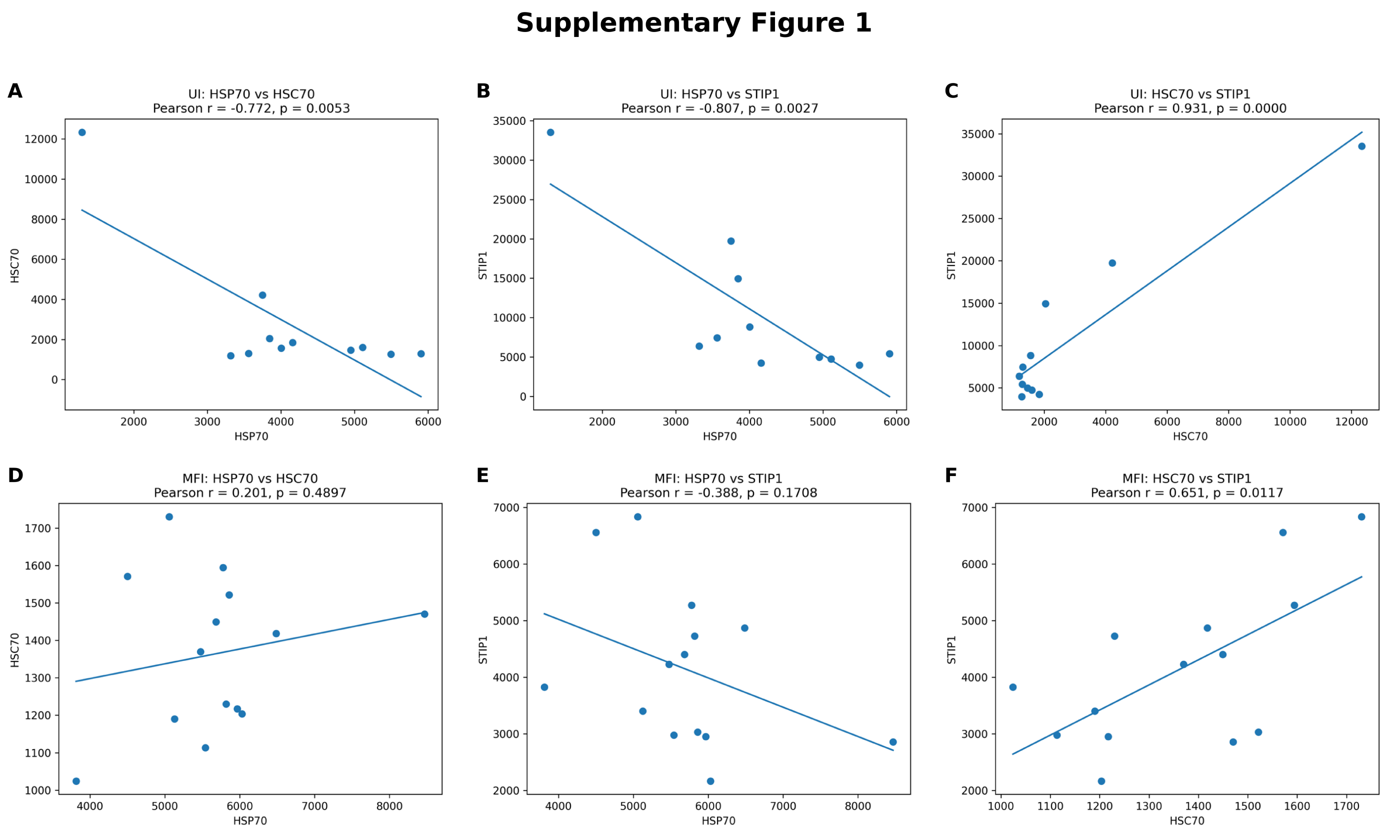

Scatterplot correlations among Heat Shock Protein 70 (HSP70), Heat Shock Cognate Protein 70 (HSC70), and Stress-Induced Phosphoprotein 1 (STIP1) in unexplained infertility (UI) and male factor infertility (MFI). Pearson correlation coefficients (r) and corresponding p-values are shown within each panel. Panels A–C represent UI correlations: (A) HSP70 vs HSC70, (B) HSP70 vs STIP1, and (C) HSC70 vs STIP1. Panels D–F represent MFI correlations: (D) HSP70 vs HSC70, (E) HSP70 vs STIP1, and (F) HSC70 vs STIP1. Regression lines indicate linear trend relationships.
